# A clinical-grade liquid biomarker detects neuroendocrine differentiation in prostate cancer

**DOI:** 10.1172/JCI161858

**Published:** 2022-11-01

**Authors:** Shuang G. Zhao, Jamie M. Sperger, Jennifer L. Schehr, Rana R. McKay, Hamid Emamekhoo, Anupama Singh, Zachery D. Schultz, Rory M. Bade, Charlotte N. Stahlfeld, Cole S. Gilsdorf, Camila I. Hernandez, Serena K. Wolfe, Richel D. Mayberry, Hannah M. Krause, Matt Bootsma, Kyle T. Helzer, Nicholas Rydzewski, Hamza Bakhtiar, Yue Shi, Grace Blitzer, Christos E. Kyriakopoulos, David Kosoff, Xiao X. Wei, John Floberg, Nan Sethakorn, Marina Sharifi, Paul M. Harari, Wei Huang, Himisha Beltran, Toni K. Choueiri, Howard I. Scher, Dana E. Rathkopf, Susan Halabi, Andrew J. Armstrong, David J. Beebe, Menggang Yu, Kaitlin E. Sundling, Mary-Ellen Taplin, Joshua M. Lang

**Affiliations:** 1 Department of Human Oncology and; 2 Carbone Cancer Center, University of Wisconsin–Madison, Madison, Wisconsin, USA.; 3 William S. Middleton Memorial Veterans Hospital, Madison, Wisconsin, USA.; 4 Department of Medicine, University of Wisconsin–Madison, Madison, Wisconsin, USA.; 5 Moores Cancer Center, University of California, San Diego, La Jolla, California, USA.; 6 Wisconsin State Lab of Hygiene, Madison, Wisconsin, USA.; 7 Lank Center for Genitourinary Oncology, Dana-Farber Cancer Institute, Boston, Massachusetts, USA.; 8 Department of Pathology and Laboratory Medicine, University of Wisconsin–Madison, Madison, Wisconsin, USA.; 9 Genitourinary Oncology Service, Department of Medicine and; 10 Biomarker Development Program, Memorial Sloan Kettering Cancer Center, New York, New York, USA.; 11 Department of Biostatistics and Bioinformatics and; 12 Duke Cancer Institute Center for Prostate and Urologic Cancers, Department of Medicine, Duke University, Durham, North Carolina, USA.; 13 Department of Biomedical Engineering and; 14 Department of Biostatistics and Medical Informatics, University of Wisconsin–Madison, Madison, Wisconsin, USA.

**Keywords:** Oncology, Prostate cancer

## Abstract

**Background:**

Neuroendocrine prostate cancer (NEPC) is an aggressive subtype, the presence of which changes the prognosis and management of metastatic prostate cancer.

**Methods:**

We performed analytical validation of a Circulating Tumor Cell (CTC) multiplex RNA qPCR assay to identify the limit of quantification (LOQ) in cell lines, synthetic cDNA, and patient samples. We next profiled 116 longitudinal samples from a prospectively collected institutional cohort of 17 patients with metastatic prostate cancer (7 NEPC, 10 adenocarcinoma) as well as 265 samples from 139 patients enrolled in 3 adenocarcinoma phase II trials of androgen receptor signaling inhibitors (ARSIs). We assessed a NEPC liquid biomarker via the presence of neuroendocrine markers and the absence of androgen receptor (AR) target genes.

**Results:**

Using the analytical validation LOQ, liquid biomarker NEPC detection in the longitudinal cohort had a per-sample sensitivity of 51.35% and a specificity of 91.14%. However, when we incorporated the serial information from multiple liquid biopsies per patient, a unique aspect of this study, the per-patient predictions were 100% accurate, with a receiver-operating-curve (ROC) AUC of 1. In the adenocarcinoma ARSI trials, the presence of neuroendocrine markers, even while AR target gene expression was retained, was a strong negative prognostic factor.

**Conclusion:**

Our analytically validated CTC biomarker can detect NEPC with high diagnostic accuracy when leveraging serial samples that are only feasible using liquid biopsies. Patients with expression of NE genes while retaining AR-target gene expression may indicate the transition to neuroendocrine differentiation, with clinical characteristics consistent with this phenotype.

**Funding:**

NIH (DP2 OD030734, 1UH2CA260389, R01CA247479, and P30 CA014520), Department of Defense (PC190039 and PC200334), and Prostate Cancer Foundation (Movember Foundation — PCF Challenge Award).

## Introduction

The majority of prostate cancers are histologically classified as adenocarcinomas. Prostate adenocarcinoma is driven by androgens, expresses prostate-specific antigen (PSA) and other androgen receptor (AR) target genes, and responds to AR-directed therapies. In contrast, neuroendocrine prostate cancer (NEPC) is an aggressive subtype of prostate cancer characterized by decreased AR signaling, increased expression of neuroendocrine markers, and an insensitivity to AR-directed therapies (1–3). NEPC can occur de novo but more commonly emerges over the course of treatment (1–3). Some studies have demonstrated that NEPC emergence can occur via transdifferentiation from a preexisting conventional adenocarcinoma (4, 5). Molecularly, NEPC is enriched for specific DNA alterations such as the loss of the key tumor suppressors RB1, TP53, and PTEN (1–3, 6). NEPC also carries distinct gene expression patterns such as decreased expression of AR target genes and increased expression of neuroendocrine markers such as synaptophysin (*SYP*) and chromogranin-A (*CHGA*) (1–3, 7). Finally, NEPC has been shown to have a distinct methylation pattern compared with prostate adenocarcinoma (2, 8, 9). The distinction between NEPC and metastatic prostate adenocarcinoma is important because the treatments are very different. Diagnosis of NEPC portends a poor prognosis with standard hormonal therapies, and patients with metastases are often treated with platinum-doublet chemotherapy similar to neuroendocrine tumors of other primary sites.

Diagnosis of NEPC is currently performed via tissue biopsies, but metastatic biopsies, in particular, can be difficult to obtain. Serial metastatic biopsies are infeasible due to the logistical challenges and the potential for complications. However, serial monitoring is required to identify an emerging NEPC phenotype. The distinct molecular features of NEPC suggest that it may be possible to identify NEPC using emerging blood-based “liquid” biopsy technology. Circulating tumor DNA–targeted (ctDNA-targeted) sequencing is now commercially available and can be used to identify multiple tumor suppressor alterations that correlate with NEPC emergence, though these alterations can also be found in adenocarcinomas (8, 10). Methylation of ctDNA is another feature that has been shown to be able to distinguish NEPC from adenocarcinoma in patients with metastases (8, 11–13). Capture and molecular profiling of circulating tumor cells (CTCs), a distinct circulating analyte, represents a complementary approach to ctDNA methylation. Past CTC studies have focused on using morphology to identify NEPC (14, 15). Multiple studies have suggested a molecular definition of NEPC (1–3, 9, 16) presenting an opportunity for a gene expression-based CTC assay (10, 17–20) that may be more specific for NEPC. A major advantage of using liquid biopsies is the ability to obtain serial sampling. The sensitivity of a single CTC or ctDNA sampling is limited because their presence and amount is influenced by the patient’s overall tumor burden and their response to treatment. If there are no CTCs or ctDNA in circulation, no assay will be able to detect anything. However, serial monitoring with liquid biopsies allows for continuous real-time assessment, including periods of both low and high tumor burden. This approach has the potential to maximize the overall sensitivity of detection, and is akin to the serial monitoring recommended for at-home rapid COVID tests; any individual test may not have the best sensitivity, but serial monitoring greatly improves this metric.

Herein, we describe a clinical-grade liquid biomarker NEPC assay for prostate cancer. We examined 116 longitudinal CTC samples from a prospectively collected institutional cohort of 17 patients with metastatic prostate cancer, baseline samples from 2 phase II trials of the AR signaling inhibitors (ARSIs) enzalutamide and abiraterone with a total of 48 patients, and 217 longitudinal samples from 91 patients from a phase II trial of the ARSI seviteronel. We have previously published on the performance and clinical characteristics of the CTC capture platform and multiplex RNA qPCR panel in metastatic prostate cancer (10, 16, 17, 21, 22). In this study, we describe the analytical validation to translate this research assay into a clinical-grade assay in a Clinical Laboratory Improvement Amendments–certified (CLIA-certified) lab at the Wisconsin State Lab of Hygiene (WSLH). We then explored the performance characteristics of this assay at detecting NEPC clinically, as well as the prognostic implications of detecting emerging neuroendocrine differentiation in CTCs.

## Results

### Analytical validation for CLIA.

**Figure 1:**
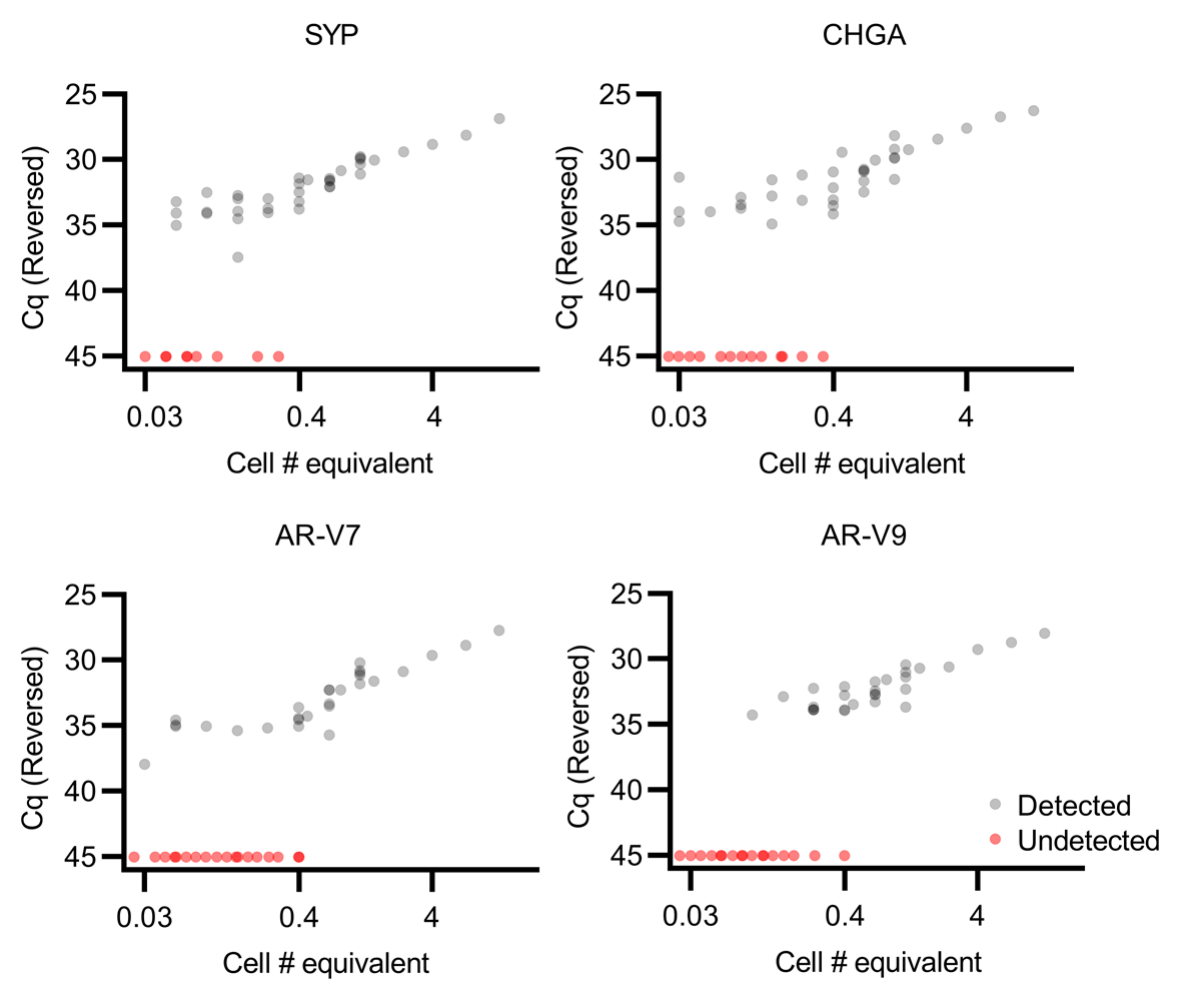
Multiplex qPCR in 22RV1 cells can detect RNA down to below a single cell. 22RV1 cell number is plotted against qPCR Cq (*n =* 47 for each gene). Four genes, SYP, CHGA, AR-V7, and AR-V9, were selected for their variable expression in CTCs from patients with metastatic prostate cancer. qPCR is able to reliably detect RNA as low as an equivalent of 0.4 cells; above this, the relationship between Cq and input amount appears to be linear.

The capture efficiency and clinical utility of our CTC capture and multiplex qPCR platform has been extensively demonstrated (10, 16, 17, 21, 22), and we sought to establish analytical validity and identify the limits of reliable quantification of the qPCR panel for the transition to a CLIA assay (23). We first examined genes in the 22RV1 cell line, which was chosen because it expresses all of the genes in the panel. RNA was extracted from cells with the CTC capture device, and after reverse transcription a range of cDNA serial dilutions were subjected to qPCR. We found that all gene expression targets were consistently detected from quantities of 22RV1 cDNA equivalent to approximately 0.4 cells or greater (Figure 1) with a linear relationship between increasing cell numbers and qPCR Cq values. Below this threshold, targets were not consistently detected. The data demonstrate the relationship between analyte quantity and consistency of detection across replicates, a measure of qualitative precision. In order to further define the limit of quantification (LOQ) threshold, we utilized a logistic regression approach (24).

**Figure 2:**
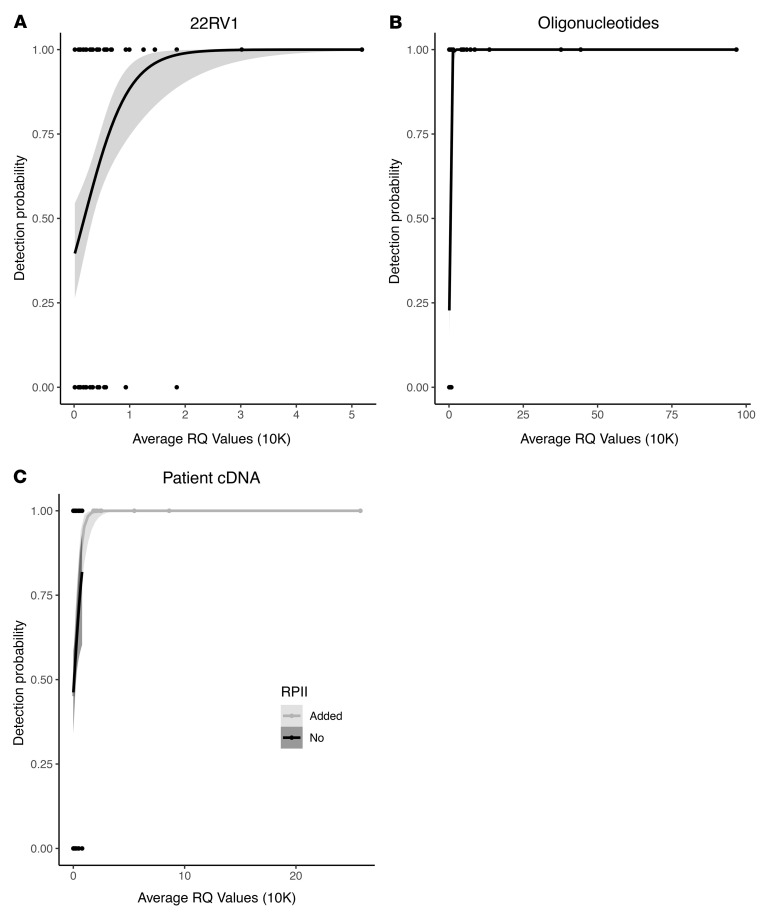
Limits of quantification for CTC multiplex qPCR. (**A**) Logistic regression demonstrating the limits of quantification using serial dilutions of 22RV1 cells (*n =* 145) including SYP, CHGA, AR-V7, and AR-V9. Five replicates were performed per gene and concentration. (**B**) Logistic regression demonstrating the limits of quantification using serial dilutions of synthetic DNA oligonucleotides (*n =* 324), including the genes SYP, CHGA, AR-V7, AR-V9, TMPRSS2, KLK3, KLK2, and FOLH1. Nine replicates were performed per gene and concentration. (**C**) Logistic regression demonstrating the limits of quantification using CTCs captured from patients with prostate cancer (*n =* 148). The same 4 genes were tested as in **A**. 4 replicates were performed per gene and sample. In addition, the ubiquitously expressed housekeeping gene RPII was added (shown in gray) for visualization purposes only because the range of relative quantity (RQ) values for the 4 genes was narrow, as expression levels were low even when detectable (shown in black). Addition of RPII allowed the visualization of the plateau region.

For 22RV1 cDNA, the LOQ threshold identified had a Cq value of 31.40 cycles across all 4 genes tested (Figure 2A). To orthogonally validate this threshold across our entire gene target panel, we utilized synthetic DNA oligonucleotides and found a similar LOQ threshold Cq value of 31.00 cycles (Figure 2B). Finally, we tested 18 patient samples using the same methodology to establish the appropriate cutoff value in clinical specimens and again identified a similar LOQ threshold Cq of 31.71 cycles (Figure 2C). All 3 experiments resulted in comparable LOQ thresholds, and so we established this clinical specimen cutoff as our LOQ going forward. For this assay, we used the molecular definition of NEPC of absent expression of the AR target genes and expression of either of the 2 neuroendocrine markers on our panel, defined by the LOQ threshold.

### NEPC diagnostic accuracy.

**Figure 3:**
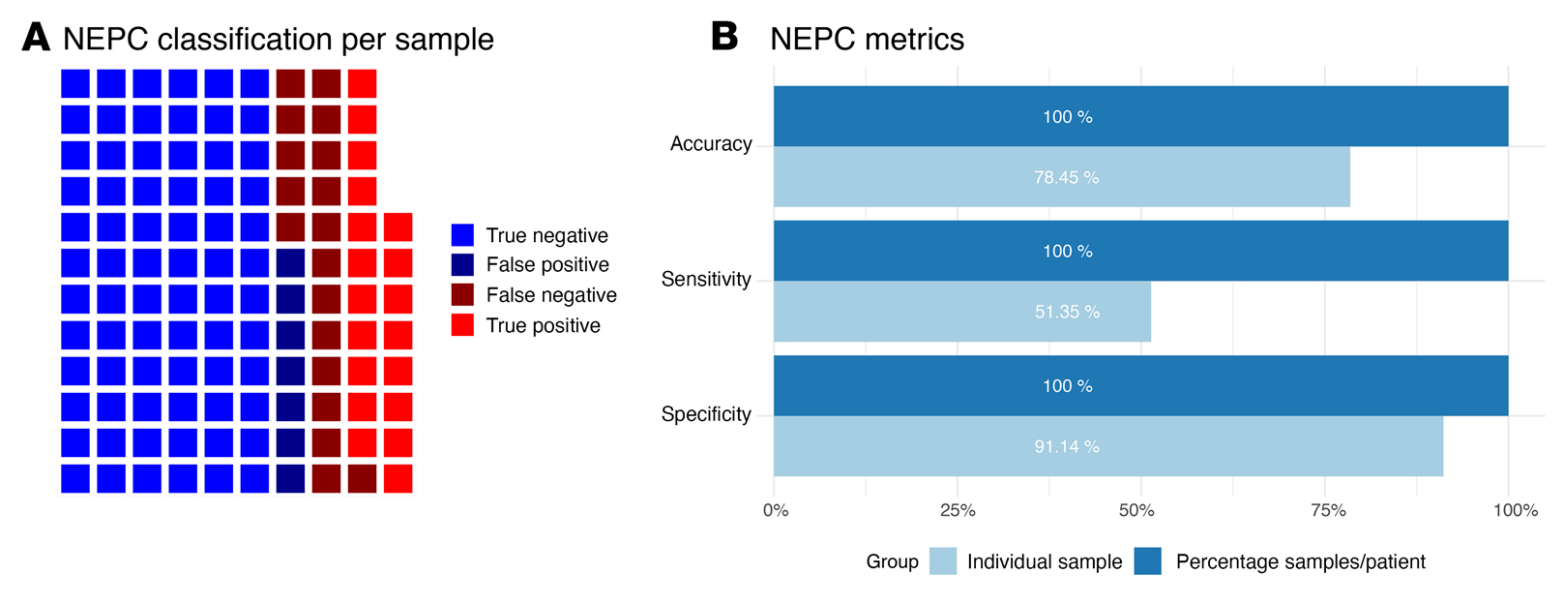
NEPC liquid biomarker performance. (**A**) Waffle plot shows the accuracy of NEPC classification on a per-sample basis across our institutional longitudinal samples. (**B**) Bar plot shows the performance metrics of NEPC classification on an individual per-sample basis versus leveraging serial sampling using liquid biomarkers to calculate the percent positive samples per patient (cutoff of 33%) from across our institutional longitudinal samples.

Using our liquid biomarker assay, we examined a prospective institutional cohort of 116 longitudinal samples (37 from tissue-confirmed patients with NEPC and 79 from adenocarcinoma cases) from 17 patients with metastatic prostate cancer (7 with NEPC and 10 with adenocarcinoma). Across all samples, our liquid biopsy approach had a sensitivity of 51.35% and a specificity of 91.14% (Figure 3, A and B). The overall accuracy was 78.45% (Figure 3, A and B). We further evaluated the specificity of the assay in baseline samples from 2 phase II ARSI clinical trials of enzalutamide (*n =* 21) and abiraterone (*n =* 27). The inclusion criteria for both trials required a diagnosis of adenocarcinoma, and our liquid biopsy assay confirmed that no patients were classified as NEPC, resulting in a specificity of 100%. Finally, we also evaluated our assay in a phase II adenocarcinoma trial (217 longitudinal samples from 91 patients) of the experimental ARSI seviteronel. When examining the baseline samples from this trial, only a single sample was inaccurately classified as NEPC using our assay, resulting in a specificity of 99%, though this patient failed treatment after only 52 days, consistent with a more aggressive disease phenotype. The high specificity across all data sets is promising, and the lower sensitivity is unsurprising on a per sample basis, given the variability in the clinical status at the time of sample collection. In the patients with NEPC, more CTCs seem to improve the odds of detection by our assay, though statistical significance was not reached due to the small number of samples with available CTC enumeration data (Supplemental Figure 1). Patients with NEPC who are responding to treatment at the time of CTC collection would be expected to have lower CTC burdens in general, thus dropping their NEPC markers below the threshold of detection. This is likely one of the main reasons why the per-patient serial sample sensitivity (shown below) improves so much over the per-sample sensitivity. Tumor burden, and thus CTC number, can fluctuate, but when we can monitor patients over time, we are much more likely to catch the times when CTC numbers are sufficient for the diagnosis of NEPC.

### Longitudinal sampling at multiple time-points improves diagnostic accuracy.

**Figure 4:**
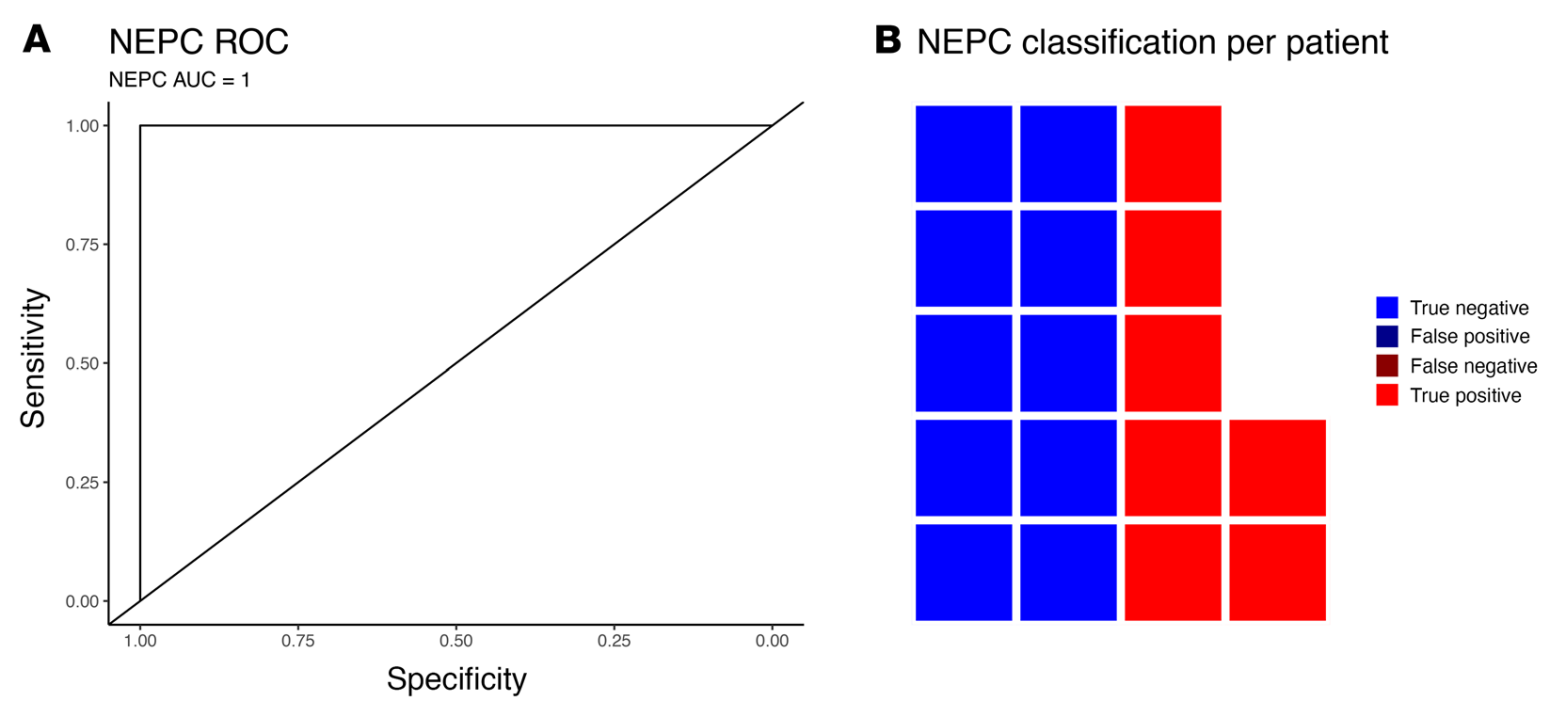
NEPC liquid biomarker serial sample performance. (**A**) ROC curve of NEPC classification accuracy leveraging serial sampling using liquid biomarkers to calculate the percent positive samples per patient across our institutional longitudinal samples. (**B**) Waffle plot shows the accuracy of NEPC classification on a per-patient basis using the percent positive across our institutional longitudinal samples.

Liquid biopsies are uniquely suited for serial sampling. For any individual sample, there will always be external factors that may affect the accuracy of an assay, such as treatment response as described above. However, serial testing can be used to minimize the effect of technical and biological artifacts and improve performance. A relevant noncancer example is COVID testing with rapid antigen tests, which also have mediocre sensitivity, but high specificity with just a single test due to fluctuating antigen levels. Serial testing in this setting is commonly used improve the sensitivity of these assays (25, 26). We sought to use a similar approach and evaluate each patient using all of their serial blood samples to calculate the percentage of positive samples instead of just 1 individual sample. Patients had a median of 7 serial CTC collections over 18.4 months. Using receiver-operating-curve (ROC) analysis to compare this with the actual NEPC status of the patient, the AUC was 1 (Figure 4A). A cutoff at 33% of the serial samples testing positive resulted in an accuracy of 100% (Figure 3B and Figure 4B). This improvement in performance using serial samples supports the use of such an approach in future clinical trials. A single snapshot in time may not accurately reflect a changing clinical picture, but continual serial molecular monitoring can improve assay performance.

Patient no. 4 with NEPC had a CT scan that showed multiple bony and visceral lesions with a PSA of 174 ng/mL. A bone biopsy was obtained that showed poorly differentiated carcinomas, which suggested metastatic prostate cancer. He started ADT, which was followed by 6 cycles of docetaxel. His first blood collection on the protocol was 6 months after the biopsy, after chemohormonal therapy, and was positive for *SYP* and absent for AR targets, consistent with NEPC. At this time, his PSA was 1.46. He continued with ADT with low PSAs, and, in 3 subsequent CTC collections, had negative *SYP* and AR target expression (consistent with his low PSA). However, after approximately 1.5 years, he developed recurrent rib pain, and CTC collection was once again positive for *SYP* as well as *CHGA*. A CT scan showed widespread metastases, and a liver biopsy demonstrated small cell prostate cancer, with diffuse positivity of *CHGA* and *SYP*. These data suggest that the patient’s early *SYP*-positive liquid biopsy after initial chemohormonal therapy likely represented at least some neuro-endocrine differentiation in a tumor that was responding to treatment. However, on ADT alone and over time, these cells were able to regrow and become fully clinically apparent NEPC, with concordant results on the liquid and solid tissue biopsies (both *CHGA*/*SYP* positive). Subsequently, the patient was started on cisplatin/etoposide, with a good clinical response, and another liquid biopsy during cycle 2 again reverted to *CHGA*/*SYP* negative. No further liquid biopsies were obtained as the patient completed chemotherapy, but he then declined and went into hospice care.

Patient no. 7 with NEPC is another interesting case study, who, over the course of 2 years, had multiple liquid biopsies that were positive for *SYP*, but were negative for *CHGA* and AR target gene expression. During this time period, he was treated on 2 clinical trials with experimental therapies, as well as abiraterone, to which he had an initial response but then progressed. There was clinical concern for NEPC due to the lack of durable response, but a bone biopsy 1 year into his time on the study was negative for *SYP*/*CHGA*. However, 7 months later, a repeat bone biopsy showed focal *SYP* positivity with *CHGA* negativity, consistent with the recurrent *SYP*-positive and *CHGA* negative CTC results. The repeated *SYP* positivity on the liquid biopsies long before even focal positivity on the bone biopsies highlights the importance of continual serial molecular monitoring of disease with liquid biopsies, as a single metastatic tissue biopsy only provides a snapshot in time and space that may not reflect a rapidly changing clinical picture.

### Identification of neuroendocrine emergence.

**Figure 5:**
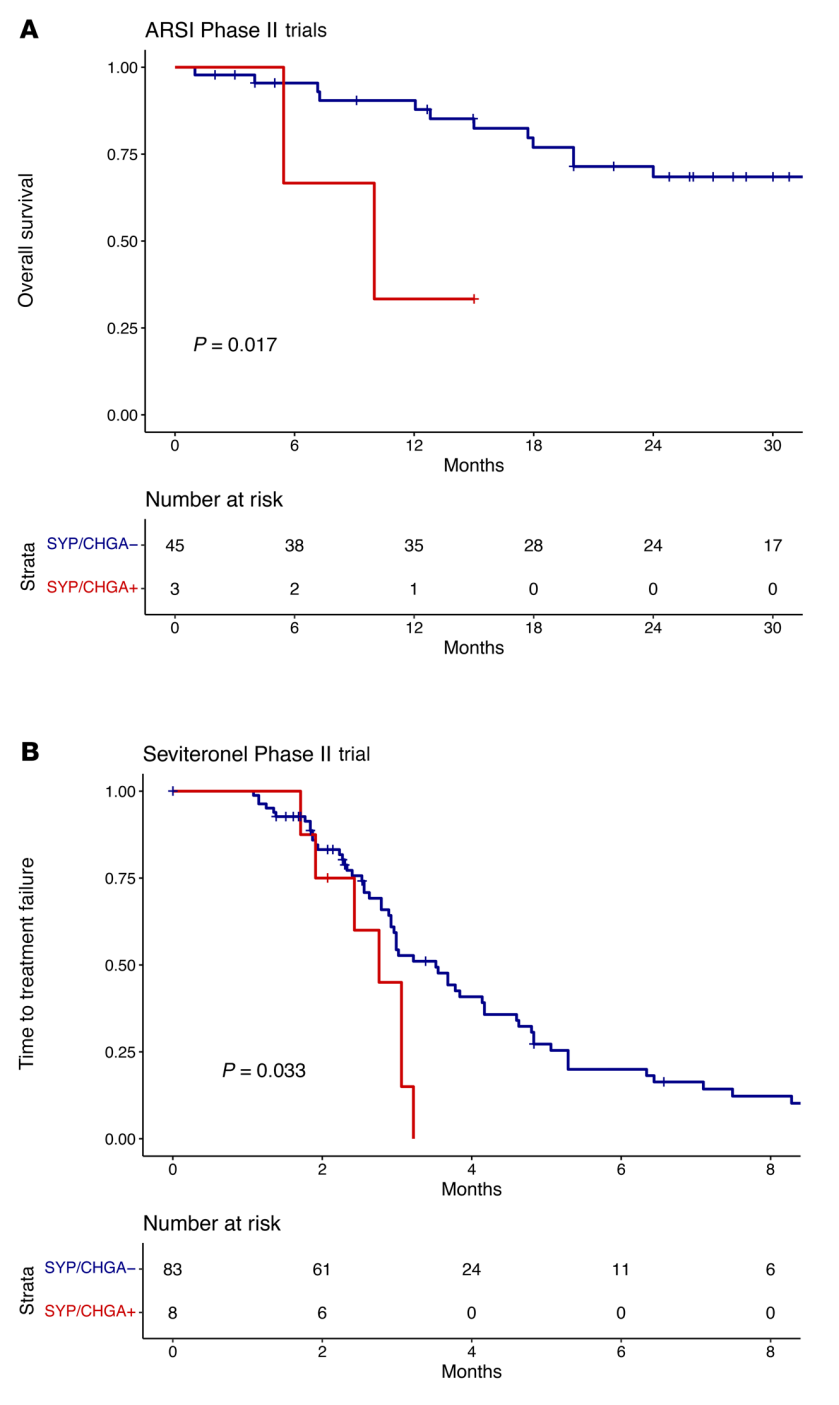
Clinical outcomes from ARSI trials and emergence of neuroendocrine markers. (**A**) Kaplan-Meier curves show that patients with expression of neuroendocrine markers, even with preserved AR target gene expression, have worse overall survival (OS) in 2 phase II adenocarcinoma ARSI (enzalutamide and abiraterone) clinical trials. *P* is calculated via log-rank test. (**B**) Kaplan-Meier curves show that patients with expression of neuroendocrine markers, even with preserved AR target gene expression, have worse time to treatment failure (TTF) in a phase II adenocarcinoma ARSI (seviteronel) clinical trial. *P* values were calculated via log-rank test.

Metastatic NEPC typically emerges under the selective pressure to ARSIs; generally, there is a transition period where a mixed population of adenocarcinoma and NEPC coexist (27). We hypothesized that this state would likely appear to have neuroendocrine expression from the NEPC component, as well as preserved AR target gene expression from the adenocarcinoma component. In addition, there are reports of atypical neuroendocrine tumor cells that coexpress AR target genes and neuroendocrine markers (27). We sought to further evaluate this in 2 phase II adenocarcinoma ARSI trials with abiraterone and enzalutamide, respectively. None of the baseline samples from these 2 trials met the above criteria for NEPC on their CTCs, consistent with their adenocarcinoma diagnosis. However, we identified 3 patients from a total of 48 total patients with *SYP* and/or *CHGA* expression in their baseline blood samples without loss of AR target gene expression. These patients had significantly worse OS (Figure 5A; hazard ratio = 5.5906 [1.143-27.36], log-rank *P =* 0.017) as would be expected by patients with emerging neuroendocrine differentiation.

We also evaluated this in the seviteronel phase II trial, in which we profiled 217 longitudinal samples from 91 patients with metastatic castration-resistant prostate cancer (mCRPC) who were progressing on first-line ARSI therapy. This is generally a poor-risk population, and the reported median progression-free survival (PFS) for patients receiving a second-line ARSI after first-line ARSI ranges from 1.7 to 3.5 months in large randomized trials (28, 29). The median time to treatment failure (TTF) in the seviteronel trial was 3.06 months, compared with a reported median PFS of 1.7 months in patients who received abiraterone — which has the same mechanism as seviteronel — after enzalutamide treatment in a British Columbia trial (29). In the seviteronel trial, only 1 patient met the CTC criteria for NEPC at baseline. However, 8 of 91 total patients demonstrated expression of the neuroendocrine markers *SYP* and/or *CHGA* in at least 1 of their blood collections, usually without concordant loss of AR target genes. These patients demonstrated significantly decreased TTF with seviteronel (Figure 5B; hazard ratio = 2.387 [1.053-5.414], log-rank *P =* 0.033). Data from these 3 ARSI trials suggest that our liquid biopsy assay could be used for screening and early detection of the transition to an aggressive mixed or atypical neuroendocrine phenotype associated with ARSI resistance. Identification of neuroendocrine emergence could influence clinical management and favor novel therapeutic strategies and chemotherapy for these patients.

## Discussion

In this manuscript, we describe a clinical-grade liquid biomarker that can accurately identify NEPC in patients with metastatic prostate cancer and detect the early emergence of neuroendocrine features associated with poor prognosis on ARSIs. In total, we profiled 381 CTC samples from 156 patients using a multiplex qPCR assay (10, 16, 17, 21, 22) from both institutional cohorts and clinical trials. We included CTC data from 68 previously published samples as well as 313 new samples first described herein. A unique aspect of our study is the longitudinal profiling throughout the disease course. Leveraging the additional information provided by serial liquid biopsies, the assay achieved a perfect NEPC ROC AUC of 1. In addition, the assay is potentially able to identify a transition to a more neuroendocrine phenotype that is associated with worse overall survival (OS) and TTF on an ARSI. The gene expression phenotype of NEPC underlying our assay has been extensively validated (1-4, 6–8, 30), and we have used these well-described transcriptional patterns to design our prostate cancer CTC assay (10, 16, 17, 21, 22).

Metastatic NEPC has a dismal prognosis and is a subtype that needs more effective treatment options. To develop novel therapeutic strategies, ongoing and future NEPC clinical trials need a way to screen and monitor patients for inclusion. Currently, NEPC is typically suspected when PSA and radiographic/clinical progression begin to diverge, at which point a tissue biopsy is typically performed and a pathologic diagnosis of NEPC can be used for treatment decisions and/or clinical trial eligibility. However, a biopsy may be falsely negative due to sampling error, or it may be nondiagnostic. If the patient continues to be clinically suspicious for neuroendocrine disease, a second biopsy is sometimes attempted, but as patients clinically decline, the window of opportunity to act rapidly disappears. An accurate neuroendocrine liquid biopsy would allow for continuous real-time screening of any patients with metastatic prostate cancer for neuroendocrine differentiation, and confirmation of a positive test can be completed with a tissue biopsy or additional liquid biopsies. Blood can be easily collected in conjunction with patients’ regular PSA and laboratory checks. The main advantage of this assay is that it can detect the emergence of neuroendocrine markers before loss of AR signaling portending a poor prognosis on ARSIs. If this is identified early, before clinically evident NEPC, this would warrant close monitoring and consideration of a change in therapy or clinical trial. In addition, our assay can be used to expand eligibility criteria and allow seamless screening and enrollment into prospective NEPC therapeutic clinical trials.

A major strength of this assay is a straightforward and well-understood CTC capture and multiplex qPCR workflow. This scalable and automated process allows for rapid turnaround time from sample collection on the order of 2–3 days. Indeed, the main rate-limiting step is the collection of a sufficient number of samples to fill up a parallel assay run. These data are the foundation for establishing this assay in the CLIA certified laboratory in the WSLH. We have conducted extensive analytical validation on the CTC multiplex qPCR approach to understand the performance characteristics and identify LOQ for this assay and developed the necessary quality monitoring tools at each step of the workflow. This research test is being transitioned to a clinically orderable CLIA test at the WSLH. Since 2016, an Investigational Device Exemption, which requires rigorous analytical validation and CLIA certification, is now required for any biomarker used to guide therapy decisions in prospective interventional clinical trials. CLIA liquid biomarkers are therefore an absolute requirement for translation from exclusively research use to clinical trials and practice.

Prospective testing of this assay is being performed currently in ongoing trials in metastatic prostate cancer (NCT02445976, NCT01942837, NCT03725761, NCT02025010, and NCT04126070), and this assay will also be integrated as a correlative biomarker in the planned biomarker-driven Alliance A032102 PREDICT trial. A liquid biomarker to detect early neuroendocrine differentiation would represent a paradigm shift in oncology, transforming the way patients with metastatic prostate cancer are monitored for the emergence of aggressive neuroendocrine disease.

## Methods

### Analytical validation and limit of quantification of multiplex qPCR.

CLIA guidelines require that the performance of a qualitative assay readout must show precision down to the lower limit of what is considered the reportable range (23). The limit of detection is defined as the cutoff where signal is no longer detectable, while the LOQ is defined as the cutoff where signal is detected with adequate precision. The LOQ is also considered the analytical sensitivity of the test system. We interrogated the LOQ of our qPCR assay in 3 different sets of experiments described below. Additional details are available in the Supplemental Methods.

### 22RV1 cell lines.

The genes SYP, CHGA, AR-V7, and AR-V9 were selected to assess the limits of quantification, as these genes have variable expression across patient samples. While the first 2 are used for NEPC detection, the latter 2 are not and were on our panel for other purposes (10). 22RV1 is a castration-resistant model that has some expression of all 4 of these genes and was chosen as the first model system. The qPCR LOQ was assessed using serial dilutions of cDNA from 22RV1 cells that had been extracted using our CTC extraction platform (10, 16, 17, 21, 22). Quantities of cDNA equivalent to a range of 0.03–12 total 22RV1 cells were evaluated in replicate to obtain a mix of detected and undetected specimens for each gene. 5 technical replicates were performed per experimental condition, which were then quantified using our multiplex qPCR panel.

### Synthetic DNA oligonucleotides.

To validate that the qPCR LOQ are broadly applicable across genes, additional genes were tested using synthetic DNA oligonucleotide sequences. The DNA oligonucleotides were expanded to include all the genes in the NEPC panel: *SYP*, *CHGA*, *AR-V7*, *AR-V9*, *TMPRSS2*, *KLK3*, *KLK2*, and *FOLH1*. The synthetic oligonucleotides serve as surrogates for RNA transcripts, since, after reverse transcription, qPCR is the same for DNA and RNA. These oligonucleotides were also subject to replicate evaluations of serial dilutions followed by multiplex qPCR to obtain a mix of detected and undetected specimens for each gene, with 9 technical replicates per experimental condition.

### Patient samples.

Finally, we assessed the LOQ in 18 patient samples, none of which overlapped with the clinical specimens used below. Samples used for analytical validation were primarily from patients responding to treatment, and thus likely with lower CTC burdens, in order to assess the lower limits of sensitivity of the assay. We used the same panel as in the 22RV1 experiment. CTCs were captured and samples were split into 4 technical replicate evaluations per patient. Multiplex qPCR was performed as above.

For logistic regression evaluation of the LOQ, different concentrations of materials were evaluated in replicates and each replicate was then categorized as either detected, 1, or not detected, 0, for any replicate with at least 1 detectable value (31). Logistic regression was then used to define the average relative quantity (RQ) value associated with the successful detection of 50% of replicates with 90% confidence (24). RQ was converted to more interpretable Cq values using the following formula: Cq = 43 – log_2_(RQ).

### Clinical validation — institutional cohort.

Between 2014 and 2020, longitudinal blood specimens from patients with metastatic prostate cancer were collected on an institutional IRB–approved prospective biospecimen protocol (1202–1214) for CTC analysis at the University of Wisconsin Carbone Cancer Center. All patients were required to have histologically confirmed metastatic prostate cancer. Eligibility for this study in particular required multiple samples from the same patient over time. In total, 116 longitudinal blood samples were collected from 17 patients with metastatic prostate cancer who were treated at the University of Wisconsin Carbone Cancer Center (UWCCC). In the UW cohort, we included all patients that were enrolled on our institutional liquid biopsy collection protocol with longitudinal sample collection, defined as at least 2 time points for patients with NEPC, and at least 3 time points for patients with adenocarcinoma. A lower threshold was used for the patients with NEPC to capture as many of these patients as possible, though all but 1 of the NEPC patients had at least 3 timepoints. This may not represent the entire patient population, as patients who rapidly declined, as well as patients who responded durably, likely were not profiled with serial liquid biopsies. Seven patients had biopsy-confirmed NEPC on a metastatic biopsy, and 10 had metastatic prostate adenocarcinoma. Of the 7 NEPC patients, 6 biopsies had small cell morphology, and 1 was described as having neuroendocrine features. Five of the 7 NEPC biopsies were positive for both *SYP* and *CHGA*, and 2 were positive for only *SYP* without *CHGA* positivity. PSA trajectories for the patients with NEPC can be found in Supplemental Figure 2. Sample collection, processing, CTC extraction, RNA isolation, and multiplex qPCR were performed for this cohort, as well as the clinical trials below, as previously published (10). Patient characteristics can be found in Supplemental Table 1. The NEPC patients were treated with a diversity of therapies over the course of their time on this study, including platinum doublets (*n =* 5), taxanes (*n =* 1), ARSIs (*n =* 2), and investigational agents (*n =* 2). Likewise, the adenocarcinoma patients also underwent a range of treatments including ARSIs (*n =* 9), taxanes (*n =* 5), radium-223 (*n =* 1), and investigational agents (*n =* 6).

### Clinical validation — enzalutamide and abiraterone phase II trials.

In addition, baseline samples from 2 phase II ARSI trials of mCRPC were profiled (longitudinal samples were not available). The ENZA-CRPC trial (ClinicalTrials.gov ID NCT01942837) was a single-arm open-label phase II study of enzalutamide. This trial was open at the Dana-Farber Cancer Institute (DFCI), University of Washington (Seattle, Washington, USA), Beth Israel Deaconess Medical Center (Boston, Massachusetts, USA), and South Shore Hospital (Weymouth, Massachusetts, USA). Patients were eligible for inclusion if they had CRPC (defined as disease progression despite a serum testosterone lower than 50 ng/dL) and progression (PSA or soft tissue/bone) defined using the Prostate Cancer Working Group 2 (PCWG2) criteria. A diagnosis of adenocarcinoma was required, and NEPC was not allowed. Patients were then treated with enzalutamide 160 mg daily until progression (per PCWG2 criteria), toxicity, or withdrawal. In total, the trial accrued 66 patients from 2013–2017, of which 21 had evaluable samples for CTCs collected at baseline. Further details can be found in the original publication of this trial (16).

The AA-CRPC trial (ClinicalTrials.gov ID NCT02025010) was a single-arm open-label phase II study of abiraterone acetate. This trial was open at DFCI and Memorial Sloan Kettering Cancer Center. Similar to AA-CRPC, patients were eligible for inclusion if they had CRPC and progression, using the same definitions as listed above. Again, a diagnosis of adenocarcinoma was required, and NEPC was not allowed. Patients were then treated with abiraterone acetate 1000 mg daily until progression (per PCWG2 criteria), toxicity, or withdrawal. In total, the trial accrued 60 patients from 2013–2017, of which 27 had evaluable samples for CTCs collected at baseline. Further details can be found in the original publication of this trial (32).

These 2 prospective phase II ARSI trials were pooled together for CTC analysis (10) for a total of 48 patients. OS was the primary clinical endpoint and was defined as date of death or last contact relative to treatment start. Patient characteristics can be found in Supplemental Table 1. Reasons for why samples were not evaluable for CTCs included patient refusal, medical contraindications, shipping issues, sample processing issues, and QC failures.

### Clinical validation — seviteronel ARSI phase II trial.

We also profiled longitudinal samples from a single-arm open-label phase II trial of the ARSI seviteronel in mCRPC that had progressed on ARSIs (ClinicalTrials.gov ID NCT02445976). Seviteronel targets CYP17A1 and inhibits androgen biosynthesis, similar in mechanism to abiraterone. While this drug did not advance to randomized trials, the trial can still serve as a valuable resource for ARSI biomarker evaluation. Patients were eligible for inclusion if they had CRPC and progression, using the same definitions as listed above. They must also be on an LHRH analogue or have undergone orchiectomy. They also had to have received prior abiraterone or enzalutamide for at least 12 weeks (or another ARSI with a similar mechanism of action). Patients were then treated with seviteronel 600 mg daily until progression (per PCWG2 criteria), toxicity, or withdrawal. In total, 217 longitudinal CTC samples (baseline, on-treatment, and end-of-treatment/study) were evaluable from 91 patients from 2015–2018. With regards to clinical outcomes, only TTF was available. TTF (33, 34) has been used as an endpoint in other metastatic prostate cancer studies (35–38). Patients were censored at the time of last blood specimen receipt if there was no indication that treatment had been stopped. Unfortunately, the drug company developing seviteronel is defunct, and thus detailed clinical data such as OS were not obtainable. Seviteronel is not the ideal drug to study mechanisms of ARSI resistance, as the current trial has yet to report its outcomes and the drug has not been FDA approved for the treatment of prostate cancer. Thus, the results should be interpreted with this caveat in mind. For all 3 ARSI trials, a new biopsy confirming adenocarcinoma was not required prior to enrollment. However, it is unlikely that patients exhibiting clinical signs suspicious for NEPC would have been enrolled on an ARSI trial.

### Statistics

All statistical testing was performed using R version 4.1.0. All statistical tests were 2-sided. A *P* value cutoff of less than 0.05 was considered significant. The NEPC liquid biomarker was evaluated using sensitivity, specificity, and accuracy. Furthermore, ROC analysis was used to compare the percent positive samples per patient with NEPC status. Kaplan-Meier curves and Cox regression were used to analyze time-to-event data. NEPC tumors are defined by low/absent AR signaling, and expression of NEPC markers as previously defined (1–3, 9, 16). The panel design was locked prior to specimen collection as liquid biopsy samples were processed fresh, and contained 4 canonical AR target genes (*KLK2*, *KLK3*, *FOLH1*, *TMPRSS2*) and 2 neuroendocrine markers (*SYP*, *CHGA*). We utilized the molecular definition of NEPC in our assay: absent expression of the AR target genes (i.e. below the LOQ threshold), and detectable expression of either of the 2 neuroendocrine markers on our panel above our LOQ threshold. Sample processing staff were blinded to any clinical information.

### Study approval

For the 3 ARSI clinical trials, IRB approval and written informed consent was obtained at each of the participating institutions. A full list of sites can be found for each trial at clinicaltrials.gov (NCT01942837, NCT02025010, NCT02445976) and in Supplemental Table 2. For the institutional cohort, IRB approval was obtained from the University of Wisconsin–Madison IRB, and written informed consent was obtained.

## Author contributions

SGZ, JMS, JLS, and JML conceptualized and designed the project. SGZ, JMS, JLS, RRM, HE, AS, ZDS, RMB, CNS, CSG, CIH, SKW, RDM, HMK, MB, KTH, NR, HB, YS, GB, CEK, DK, XXW, JF, NS, MS, PMH, WH, HB, TKC, HIS, DER, SH, AJA, DJB, MY, KES, MET, JML acquired, analyzed, interpreted data, and drafted or revised the manuscript for intillectual content. All authors approved the final version of the manuscript. The order of cofirst authorship was determined based on SGZ leading the overall data analysis and writing. JMS led acquisition and analysis of the institutional cohort and clinical trial data. JLS led the analytical validation work.

## Supplementary Material

Supplemental data

Trial reporting checklists

ICMJE disclosure forms

## Version 1 11/01/2022

Electronic publication
