# Supplementary material for: A clinical-grade liquid biomarker detects neuroendocrine differentiation in prostate cancer
Source: J Clin Invest. 2022 Nov 1;132(21):e161858. doi: 10.1172/JCI161858 (PMC9621140; doi:10.1172/JCI161858)
Supplement: Supplemental data [file jci-132-161858-s112.pdf]

SUPPLEMENTAL METHODS – CONFLICT OF INTEREST STATEMENT (COI)

RRM serves as a consultant and an advisory board member for Astrazeneca, Aveo, Bayer, Bristol-Myers Squibb, Calithera, Caris, Dendreon, Exelixis, Janssen, Pfizer, Merck, Novartis, Sanofi, and Tempus. RRM also receives institutional research funding from Bayer and Pfizer. CEK performs a consulting or advisory role with Exelixis and receives research funding from Sanofi, AVEO, EMD Serono, Janssen Oncology, and Sanofi. TKC receives support for research, advisory board membership, consultancy, and honoraria from AstraZeneca, Aravive, Aveo, Bayer, Bristol Myers-Squibb, Calithera, Circle Pharma, Eisai, EMD Serono, Exelixis, GlaxoSmithKline, IQVA, Infinity, Ipsen, Jansen, Kanaph, Lilly, Merck, Nikang, Nuscan, Novartis, Pfizer, Roche, Sanofi, Surface Oncology, Takeda, Tempest, Up-To-Date, and CME events (Peerview, OncLive, MJH, and others). TKC also has institutional patents filed on molecular mutations and immunotherapy response and ctDNA (U.S. patent application no. 16/475,574 and U.S. provisional patent application no. 63/093,616), as well as equity in Tempest, Pionyr, Osel, and NuscanDx, and serves on committees for the National Comprehensive Cancer Network, GU Steering Committee, American Society of Clinical Oncology/European Society of Medical Oncology, and Academic & Community Cancer Research United. TKC has mentored several nonUS citizens on research projects with potential funding (in part) from non-US sources and/or foreign components. JML holds stock or other ownership interests in Salus Discovery LLC, which has licensed technology used within this study. SGZ has pending patent applications on molecular signatures in prostate cancer unrelated to this work licensed to Veracyte (U.S. patent publication nos. US20190218621A1, US2021130902A1, and US2021317531A1). SGZ's spouse is an employee of Exact Sciences and has been compensated with restricted stock units. WH is a cofounder of PathomIQ and owns equity in this company. MET is on the advisory board and receives honoraria from Janssen, Bayer, Astellas, AstraZeneca, Abbvie, Arcus Biosciences, Myvant, Roviant, and Blue Earth. XXW recieves advisory board honoraria from Novartis and research support (institutional) from Bristol-Myers Squibb. DJB holds equity in Bellbrook Labs LLC, Tasso Inc., Salus Discovery LLC (which has licensed technology used within this study), Lynx Biosciences Inc., Stacks to the Future LLC, Flambeau Diagnostics LLC, and Onexio Biosystems LLC. HE is an advisory board member for Bristol-Myers Squibb, Exelixis, Cardinal Health, and Seattle Genetics, and receives research funding from Bristol-Myers Squibb, Exelixis, Replimune, Merck, Calithera, and Roche/Genentech. HIS is a compensated consultant for/advisor to Ambry Genetics Corp., Konica Minolta Inc., Bayer, Pfizer Inc., Sun Pharmaceuticals, WCG Oncology and an uncompensated consultant/advisory to Amgen, Janssen Research & Development LLC, and Janssen Biotech Inc. HIS receives reviewers fees from Elseiver Ltd. and honoraria from Arsenal Capital; has received research funding (to his institution) from AIQ Solutions, Epic Sciences, Illumina Inc., Janssen, Menarini Silicon Biosystems, Prostate Cancer Foundation, and Thermo Fisher Scientific; and is supported in part by SPORE in Prostate Cancer grant P50 CA092629 (Memorial Sloan Kettering Cancer Center). HIS also has intellectual property rights with BioNTech, Elucida Oncology, MaBVAX, and Y-mAbs Therapeutics Inc., and nonfinancial support from Amgen, Bayer, Epic Sciences, Promontory Therapeutics (formerly Phosplatin), Pfizer Inc., Prostate Cancer Foundation, and WCG Oncology.

SUPPLEMENTAL METHODS – ANALYTICAL VALIDATION

Cell Line

The human CRPC cell line 22RV1 was maintained in cell culture flasks in 10% FBS with 5% penicillin/streptomycin in RPMI at 37C with 5% CO_2. 22RV1 22Rv1 (ATCC Cat# CRL-2505, RRID:CVCL_1045) were a gift from Scott Dehm and were authenticated in 2017 by the Wisconsin Translational Research Initiatives at the Pathology Laboratory at the University of Wisconsin. In order to count cell line cells prior to mRNA extraction, cells were first stained with Calcein AM by incubating them for 15 minutes at 37°C in 1:1000 Calcein AM in PBS. Cells were then washed and counted with a hemocytometer to obtain 100 total cells. After cells were incubated for 30 minutes at 4°C with antibody-coated PMPs, they were placed in an imaging chamber constructed of a silicone isolator (EMS Diasum, #70348-44) adhered to a glass coverslip (Chemglass, Life Sciences, #CLS17622575) for exact enumeration using fluorescence microscopy imaging. Each imaging chamber (~7x7mm^2) was imaged at 10x magnification on an inverted fluorescence microscope with an automated XYZ stage (Nikon Instruments, Inc) using a snaking grid-like pattern of smaller image tiles (~1mm^2). Uniform focus was maintained across the imaging field with NIS Elements Perfect Focus System (PFS). The imaging chamber was then placed on the ExtractMax platform (Gilson Inc., and Salus Discovery, LLC) for cell capture, using a 4 slide FlexWell tray (EMS diasum) to stabilize the chamber. Cells were pulled from the imaging chamber into the lysis well, then a second picture was acquired of the original imaging chamber to determine the exact quantity of cells effectively captured and lysed with the ExtractMax. NIS-Elements AR Microscope Imaging Software (RRID:SCR_014329, Nikon Instruments, USA)binary thresholding algorithms were used to automatically identify and count the number of cells in each image. After reverse transcription, the cDNA was then serially diluted to equivalents of 12, 7, 4, 2.5, 1.5, 1.2, 0.9, 0.7, 0.5, 0.4, 0.3, 0.2, 0.1, 0.06, and 0.03 cells, then split into separate replicate aliquots for downstream PreAmp and qPCR.

Automated mRNA Extraction

Prior to use, mRNA extraction plates and strips (Gilson, Inc) were briefly submerged in RNAse away (Life Tech), rinsed with nuclease-free distilled water (Thermofisher Scientific), and allowed to air dry. mRNA was extracted from captured CTCs by incubating them for 5 minutes at room temperature in 10% Lysis Binding Buffer (LBB) (Dynabeads mRNA DIRECT, Thermofisher Scientific) diluted in PBS containing 10uL Dynabeads Oligo(dT)25 beads (washed once with 10% LBB prior to use). The PMPs were collected then transferred through one sequential wash of wash buffer A (WBA) and one of 1% PBST then eluted into molecular grade nuclease-free water (Promega, Inc). Additional details can be found in a prior publication using this method(1).

Oligonucleotide Standards

DNA oligonucleotides (oligos) were designed as DNA sequences specific to the amplified region of each of the primer pairs included in the multi-target panel to serve as contrived sample material to evaluate the performance of the PreAmp and qPCR steps of the assay. All DNA oligos were designed by Thermo Fisher Scientific and purchased from Integrated DNA Technologies then prepared at 100 uM in TE buffer and stored at -20°C.
Several different DNA Oligo serial dilutions were prepared on different days to obtain a balance of replicate sets with a mixture of replicates with both detected and undetected signal. The different uM concentrations of the different serial dilution levels were as follows: SYP (1E-12, 1E-13, 1E-14, 1E-15, 1E-16, 1E-17); CHGA (1E-10, 1E-11, 1E-12, 1E-13, 1E-14); AR-V7 (1E-10, 1E-11, 1E-12, 1E-13, 1E-14, 1E-15); AR-V9 (1E-9, 1E-10, 1E-11, 1E-12, 1E-13, 1E-14); KLK2 (1E-10, 1E-11, 1E-12, 1E-13, 1E-14, 1E-15); KLK3 (1E-10, 1E-11, 1E-12, 1E-13, 1E-14, 1E-15): TMPRSS2 (1E-10, 1E-11, 1E-12, 1E-13, 1E-14, 1E-15); FOLH1 (1E-10, 1E-11, 1E-12, 1E-13, 1E-14, 1E-15)

CHGA:
5’CTTGAGGCGGGCCTGCCCCTCCAGGTCCGAGGCTACCCCGAGGAGAAGAAAGAGGAGGAGGGCAGC
GCAAACCGCAGACCAGAGGACCAGGAGCTGGAGAGCCTGTCGGCCATTGAAGCAGAGCTGGAGAAAGT
GGCCCACCAGCTGCAGGCACTACGGCGGGGCTGAGACACCGGC 3’

SYP:
5’GGATTGTGCCAACAAGACCGAGAGTGACCTCAGCATCGAGGTCGAGTTCGAGTACCCCTTCAGGCTGC
ACCAAGTGTACTTTGATGCACCCACCTGCCGAGGGGGCACCACCAAGGTCTTCTTAGTT 3’

AR-V7:
5’GATAAATTCCGAAGGAAAAATTGTCCATCTTGTCGTCTTCGGAAATGTTATGAAGCAGGGATGACTCTGG GAGAAAAATTCCGGGTTGGCAATTGCAAGCATCTCAAAATGACCAGACCCTGAAGAAAGGCTGACTTGCC TCATTCAAAATGAGG 3’

AR-V9:
5’AGCAGTGATCTCGGAAATGTTATGAAGCAGGGATGACCCGCGTGCGAGATAAAAATACTAGGTAACTAG AGGGACTGCGACGTTCTAAACGTTGGCTGGGAGACAACTTACTCCGTCAGAAGCGCCATCCAGGATCAC GTTACCCCGAAAAAGACATTTGCACATCTTTTGGGATCAAAGATATCA 3’

KLK3:
5'AGCCCCAAGCTTACCACCTGCACCCGGAGAGCTGTGTCACCATGTGGGTCCCGGTTGTCTTCCTCACC CTGTCCGTGACGTGGATTGGTGCTGCACCCCTCATCCTGTCTCGGATTGTGGGAGGCTGGGAGTGCGAG AAGCATTCCCAACCCTGGCAGGTGCTTGT 3'

TMPRSS2:
5'CTTACAGACCAGGAGTGTACGGGAATGTGATGGTATTCACGGACTGGATTTATCGACAAATGAGGGCAG ACGGCTAATCCACATGGTCTTCGTCCTTGACGTCGTTTTACAAGAAAACAATGGGGCTGGTTTT 3'

FOLH1:
5’AGATTTTCAACACATCATTATTTGAACCACCTCCTCCAGGATATGAAAATGTTTCGGATATTGTACCACCT TTCAGTGCTTTCTCTCCTCAAGGAATGCCAGAGGGCGATCTAGTGTATGTTAACTATGCACGAACTGAAGA CTTCTTTAAATTGGAACGGGACATGAAAATCA 3’

KLK2:
5’GATGGGCACACTGTGGGGGTGTCCTGGTGCACCCCCAGTGGGTGCTCACAGCTGCCCATTGCCTAAAG AAGAATAGCCAGGTCTGGCTGGGTCGGCACAACCTGTTTGAGCCTGAAGACACAGGCCAGAGGGTCCCT GTCAGCCACA 3’

Patient Sample Processing

Peripheral blood was collected from patients with metastatic prostate cancer under University of Wisconsin IRB approved protocol (2014-1214)(2). The protocol allowed a maximum of 50mL of blood collected in a single blood draw using EDTA vacutainers (BD Biosciences) and processed within 24 hours of collection allowing for duplicate samples for validation studies. Equivalency of mRNA yield was confirmed between blood processed immediately after draw compared to blood processed after overnight incubation at room temperature. 15 mL of blood was used for CTC isolation. Whole blood was diluted 1:1 with phosphate buffered saline (PBS, Hyclone) and 30mL of diluted blood was underlaid with 10mL of ficoll-paque PLUS (GE Healthcare) per 50mL conical tube. The blood was centrifuged for 20 minutes at 974g and harvested peripheral blood mononuclear cells (PBMCs) were washed twice with 50mL PBS. PBMCs were then depleted of CD45+ cells with standard LS columns per manufacturer’s instructions (Miltenyi Biotec). The samples were divided into replicates after CTC capture isolation and RNA extraction, but before pre-amplification.

Paramagnetic Particle (PMP) Preparation

Streptavidin coupled PMPs (Sera-Mag Streptavidin-Blocked SpeedBeads, Cytiva) at a concentration of 50ug per reaction were used for all experiments. The PMPs were washed with 0.1%Tween-20 in PBS (0.1%PBST) prior to use then incubated under agitation with 1uL each of capture antibodies against EpCAM (R&D Systems, TROP1), MUC1 (Biolegend, 16A), and TROP2 (R&D Systems, AF650), biotinylated according to the manufacturer’s recommendations (Dynabeads FlowComp Flexi Kit, Thermofisher Scientific). Prior to use, bead conjugates were washed and held in 10% FBS. Additional details can be found in prior publications using this method(1, 3).

CTC Isolation

CD45 depleted PBMCs were incubated on a rotator with antibody-labelled PMPs in a buffer containing 10%FBS for 30 minutes at 4°C with constant rotation. Cells bound to PMPs were isolated using automated ESP technology on the ExtractMax platform (Gilson Inc., and Salus Discovery, LLC) as described previously(2, 4). Briefly, PMP-bound cells were moved with magnetic manipulation through three 10% FBS wash wells before cell lysis for mRNA extraction.

RT-qPCR

The eluted mRNA sample was reverse transcribed using the high-capacity RNA to cDNA Reverse Transcription Kit (Thermofisher Scientific) according to the manufacturer's directions. cDNA was combined with TaqMan PreAmp master mix (Thermofisher Scientific) and TaqMan primers diluted 1:100 in TE buffer (Promega), and then subjected to 10 cycles of pre-amplification (PreAmp) according to the manufacturer's directions. The amplified product was then diluted 1:5 in TE buffer and combined with iTaq Universal Probes Supermix (Bio-Rad Laboratories) and the commercial TaqMan primers for these genes for 45 cycles of qPCR (CFX Connect Real-Time PCR Detection System, Bio-Rad Laboratories). The primers for AR-V9 were not commercially available but have been published previously(2, 5).

SUPPLEMENTAL REFERENCES

1. Pezzi HM, Guckenberger DJ, Schehr JL, Rothbauer J, Stahlfeld C, Singh A, et al. Versatile exclusion-based sample preparation platform for integrated rare cell isolation and analyte extraction. *Lab Chip.* 2018;18(22):3446-58.
2. Sperger JM, Emamekhoo H, McKay RR, Stahlfeld CN, Singh A, Chen XE, et al. Prospective Evaluation of Clinical Outcomes Using a Multiplex Liquid Biopsy Targeting Diverse Resistance Mechanisms in Metastatic Prostate Cancer. *J Clin Oncol.* 2021:JCO2100169.
3. Pezzi HM, Niles DJ, Schehr JL, Beebe DJ, and Lang JM. Integration of Magnetic Bead-Based Cell Selection into Complex Isolations. *ACS Omega.* 2018;3(4):3908-17.
4. Casavant BP, Guckenberger DJ, Beebe DJ, and Berry SM. Efficient sample preparation from complex biological samples using a sliding lid for immobilized droplet extractions. *Anal Chem.* 2014;86(13):6355-62.
5. Kohli M, Ho Y, Hillman DW, Van Etten JL, Henzler C, Yang R, et al. Androgen Receptor Variant AR-V9 Is Coexpressed with AR-V7 in Prostate Cancer Metastases and Predicts Abiraterone Resistance. *Clin Cancer Res.* 2017;23(16):4704-15.

SUPPLEMENTAL FIGURES

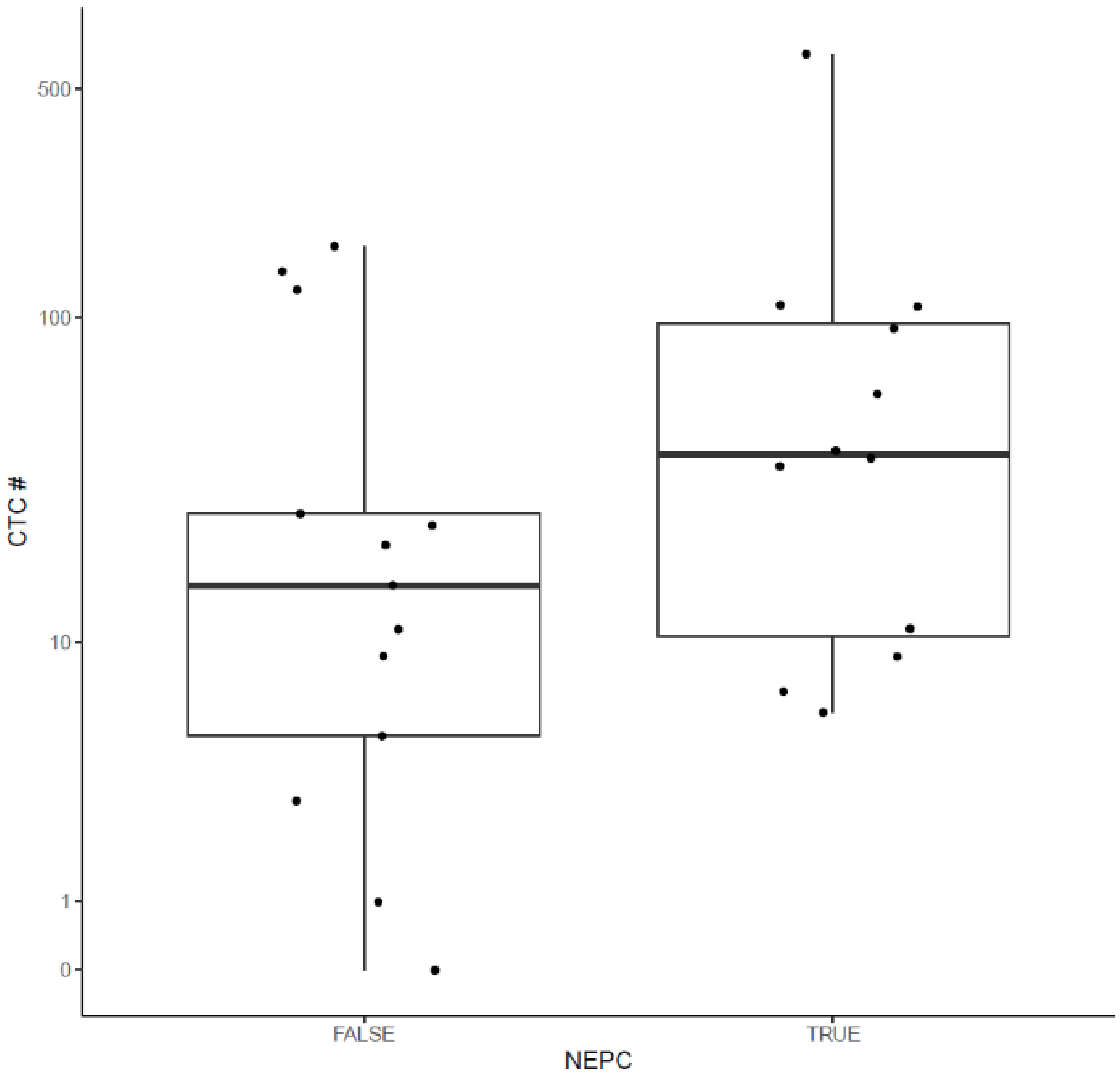

Supplemental Figure 1: In a subset of the serial samples from the NEPC patients in our institutional cohort, we had enumeration data. When we plot the per-sample NEPC CTC prediction vs. the enumeration data, we can see higher CTC numbers in the NEPC-positive compared to the NEPC-negative samples.

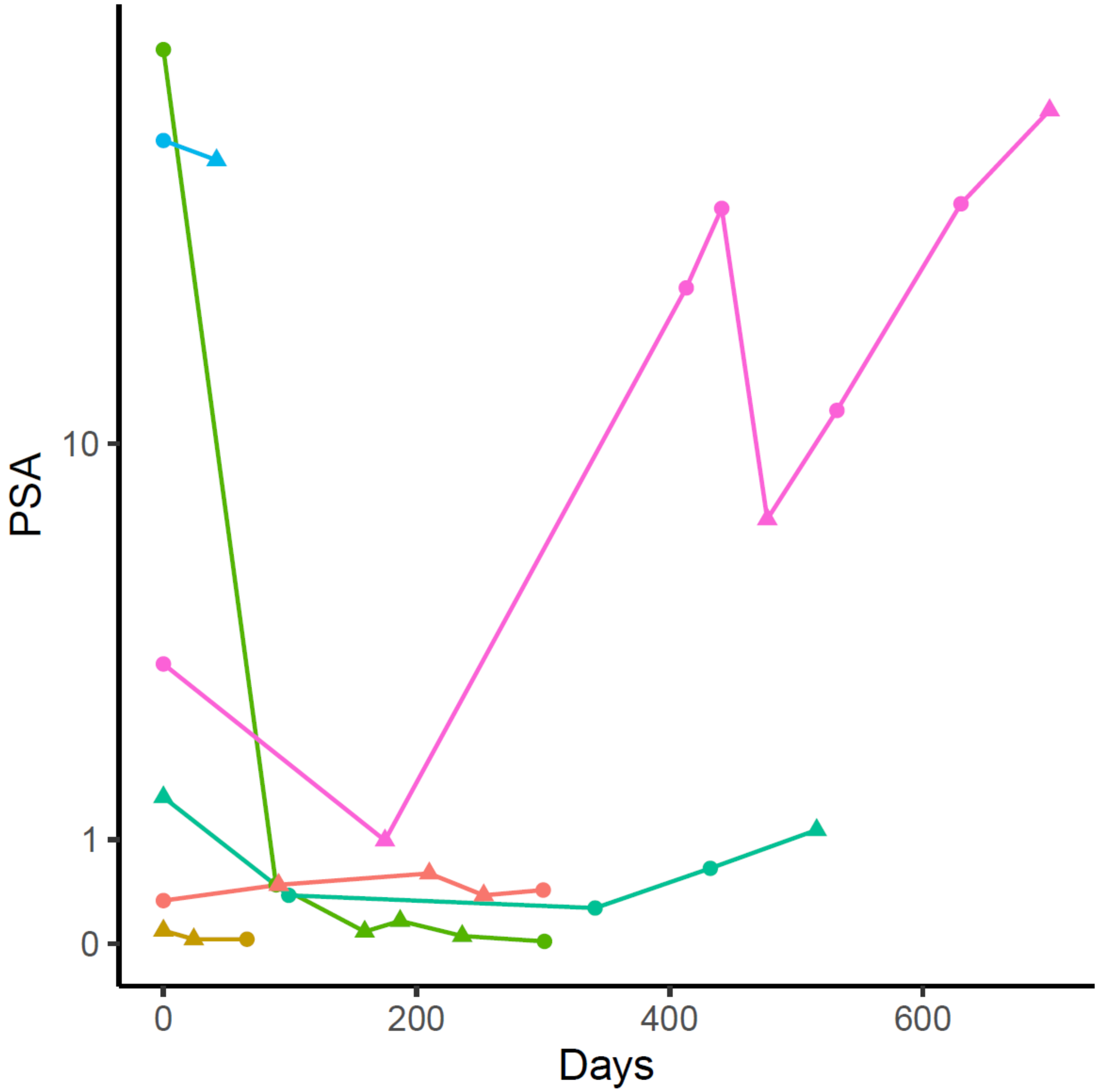

Supplemental Figure 2: PSA trends for the NEPC patients in our institutional cohort. Each sample is represented as a point, and a triangle indicates that the CTC molecular profiling suggested NEPC per the criteria described. Note that there were some missing PSA levels, primarily due to treating clinicians stopping PSA measurements once it was clear that PSA was no longer informative.

SUPPLEMENTAL TABLES

Supplemental Table 1

	UWCCC	ENZA-CRPC	AA-CRPC
Number patients	17	21	27
Age, year, median (IQR)	68 (63-78)	68 (62-72)	66 (59-74)
Race*			
White	17 (100%)	12 (57.1%)	25 (92.6%)
Black or African American	0 (-)	2 (9.5%)	0 (-)
Asian	0 (-)	1 (4.8%)	0 (-)
Unknown	0 (-)	6 (28.6%)	2 (7.4%)
ECOG performance status			
0	10 (58.8%)	14 (66.7%)	20 (74.1%)
1	2 (11.8%)	7 (33.3%)	7 (25.9%)
NA	5 (29.4%)	0 (-)	0 (-)
PSA level, ng/mL, median (IQR)	12 (3-41)	24 (8-141)	13 (7-48)
<10	7 (41.2%)	8 (38.1%)	9 (33.3%)
10-20	2 (11.8%)	1 (4.8%)	7 (25.9%)
≥20	8 (47.1%)	12 (57.1%)	10 (37.0%)
NA	0 (-)	0 (-)	1 (3.7%)
Gleason score			
≤6	3 (17.6%)	3 (14.3%)	1 (3.7%)
7	4 (23.5%)	7 (33.3%)	11 (40.7%)
8-10	6 (35.3%)	10 (47.6%)	13 (48.1%)
NA	4 (23.5%)	1 (4.8%)	2 (7.4%)
Metastases at diagnosis	7 (41.2%)	4 (19.0%)	8 (29.6%)
Prior abiraterone acetate	1 (5.9%)	1 (4.8%)	0 (-)
Prior enzalutamide**	0 (-)	0 (-)	4 (14.8%)
Prior chemotherapy	3 (17.6%)	10 (47.6%)	11 (40.7%)
Prior ketoconazole	1 (5.9%)	2 (9.5%)	2 (7.4%)
Prior sipuleucel-T	1 (5.9%)	5 (23.8%)	6 (22.2%)
Sites of metastasis			
Bone	16 (94.1%)	19 (90.5%)	0 (-)
Liver	5 (29.4%)	0 (-)	1 (3.7%)
Lung	3 (17.6%)	5 (23.8%)	0 (-)
Lymph Nodes	12 (70.6%)	11 (52.4%)	7 (25.9%)
Others	3 (17.6%)	3 (14.3%)	0 (-)

Values are n (%) or median (IQR).

*Patient determined.

**Prior enzalutamide and abiraterone excluded from ENZA-CRPC and AA-CRPC, respectively.

Supplemental Table 2

**ENZA-CRPC (NCT01942837)**	
Brigham and Women's Hospital	Boston, Massachusetts, United States, 02115
Dana-Farber Cancer Institute	Boston, Massachusetts, United States, 02115
Beth Israel Deaconess Medical Center	Boston, Massachusetts, United States, 02215
South Shore Hospital	Weymouth, Massachusetts, United States, 02190
University of Washington Medical Center/Seattle Cancer Care Alliance	Seattle, Washington, United States, 98195
**AA-CRPC (NCT02025010)**	
Brigham and Women's Hospital	Boston, Massachusetts, United States, 02115
Dana-Farber Cancer Institute	Boston, Massachusetts, United States, 02115
Memorial Sloan Kettering Cancer Center Basking Ridge	Basking Ridge, New Jersey, United States, 07920
Memorial Sloan Kettering Cancer Center Commack	Commack, New York, United States, 11725
Memorial Sloan Kettering Cancer Center West Harrison	Harrison, New York, United States, 10604
Memorial Sloan-Kettering Cancer Center	New York, New York, United States, 10065
Memorial Sloan Kettering Cancer Center Rockville Centre	Rockville Centre, New York, United States, 11510
Memorial Sloan Kettering Cancer Center Sleepy Hollow	Sleepy Hollow, New York, United States, 10591
**Seviteronel trial (NCT02445976)**	
University of Alabama	Birmingham, Alabama, United States, 35249
Mayo Clinic	Scottsdale, Arizona, United States, 85054
University of California at Los Angeles	Los Angeles, California, United States, 90095
Yale University	New Haven, Connecticut, United States, 06519
Mayo Clinic - Jacksonville	Jacksonville, Florida, United States, 32224
Moffitt Cancer Center	Tampa, Florida, United States, 33612
Tulane University	New Orleans, Louisiana, United States, 70112
Beth Israel Deaconess Medical Center	Boston, Massachusetts, United States, 02215
Dana-Farber Cancer Institute	Boston, Massachusetts, United States, 02215
Karmanos Cancer Institute	Detroit, Michigan, United States, 48201
University of Minnesota	Minneapolis, Minnesota, United States, 55455
Washington University	Saint Louis, Missouri, United States, 63110
GU Research Network	Omaha, Nebraska, United States, 68130
New Mexico Cancer Care Alliance	Albuquerque, New Mexico, United States, 87106
Memorial Sloan Kettering Cancer Center	New York, New York, United States, 10065
University of North Carolina	Chapel Hill, North Carolina, United States, 27514
Carolina Urologic Research Center	Myrtle Beach, South Carolina, United States, 29572
University of Virginia	Charlottesville, Virginia, United States, 22903
Virginia Oncology Associates	Hampton, Virginia, United States, 23666
University of Washington	Seattle, Washington, United States, 98109
University of Wisconsin Carbone Cancer Center	Madison, Wisconsin, United States, 53715
